# Response assessment after stereotactic body radiation therapy for spine and non-spine bone metastases: results from a single institutional study

**DOI:** 10.1186/s13014-022-02004-7

**Published:** 2022-02-21

**Authors:** Dora Correia, Barbara Moullet, Jennifer Cullmann, Rafael Heiss, Ekin Ermiş, Daniel M. Aebersold, Hossein Hemmatazad

**Affiliations:** 1 Department of Radiation Oncology, Inselspital, Bern University Hospital and University of Bern, Bern, Switzerland; 2 Centre for Proton Therapy, Paul-Scherrer Institute, Villigen, Switzerland; 3 Department of Radiation Oncology, Triemli Hospital Zürich, Zürich, Switzerland; 4 Department of Radiology, Inselspital, Bern University Hospital and University of Bern, Bern, Switzerland

**Keywords:** Spine metastases, SBRT, Response assessment, Pain response

## Abstract

**Background:**

The use of stereotactic body radiation therapy (SBRT) for tumor and pain control in patients with bone metastases is increasing. We report response assessment after bone SBRT using radiological changes through time and clinical examination of patients.

**Methods:**

We analyzed retrospectively oligo-metastatic/progressive patients with bony lesions treated with SBRT between 12/2008 and 10/2018, without in-field re-irradiation, in our institution. Radiological data were obtained from imaging modalities used for SBRT planning and follow-up purposes in picture archiving and communication system and assessed by two independent radiologists blind to the time of treatment. Several radiological changes were described. Radiographic response assessment was classified according to University of Texas MD Anderson Cancer Center criteria. Pain response and the neurological deficit were captured before and at least 6 months after SBRT.

**Results:**

A total of 35 of the 74 reviewed patients were eligible, presenting 43 bone metastases, with 51.2% (n = 22) located in the vertebral column. Median age at the time of SBRT was 66 years (range 38–84) and 77.1% (n = 27) were male. Histology was mainly prostate (51.4%, n = 18) and breast cancer (14.3%, n = 5). Median total radiation dose delivered was 24 Gy (range 24–42), in three fractions (range 2–7), prescribed to 70–90% isodose-line. After a median follow-up of 1.8 years (range < 1–8.2) for survivors, complete or partial response, stable, and progressive disease occurred in 0%, 11.4% (n = 4), 68.6% (n = 24), and 20.0% (n = 7) of the patients, respectively. Twenty patients (57.1%) died during the follow-up time, all from disease progression, yet 70% (n = 14) from this population with local stable disease after SBRT. From patients who were symptomatic and available for follow-up, almost half (44.4%) reported pain reduction after SBRT.

**Conclusions:**

Eighty percent of the patients showed local control after SBRT for bone metastases. Pain response was favorable. For more accurate response assessment, comparing current imaging modalities with advanced imaging techniques such as functional MRI and PET/CT, in a prospective and standardized way is warranted.

*Trial registration* Retrospectively registered.

## Background

Many patients suffering from solid tumors develop metastatic cancer with single, limited or diffuse metastases. Besides lung and liver, bone is a common site of metastasis [1]. Caused in up to 70% by prostate and breast cancer, bone metastases are a major cause for morbidity [2]. Bone metastases are predominantly located in the vertebral column and it is estimated that over 10% of cancer patients develop symptoms at this site [3, 4].

The role of radiotherapy in palliating pain for bone metastases is well established [5]. In the past, patients with painful bone metastases had a limited median overall survival (OS) of 7–9 months [6–8]. However, patients show increased OS in recent years due to improved treatment approaches, thus being essential to provide a highly effective local therapy. SBRT is a promising modality to treat bone metastases with locally ablative intent [9] and has been used frequently in daily practice for more than a decade. Nevertheless, the results of prospective randomized trials comparing conventional radiotherapy to SBRT are very recent [10–12]. The pain response is the focus of these prospective randomized trials and none of them has reported the radiological response assessment yet. As histological confirmation is challenging and costly in case of suspicious tumor progression after SBRT, an accurate radiological assessment is of utter importance and could avoid unnecessary interventions in asymptomatic patients. The SPIne response assessment in Neuro-Oncology (SPINO) group consensus uniforms the various criteria for radiological assessment of therapy response after spinal SBRT [13]. Still, few studies have evaluated the detailed radiological changes in bone metastases after SBRT [14–16]. For bone metastases, there are specific aspects to consider in the interpretation of radiological changes after SBRT, including pseudo-progression, vertebral compression fracture (VCF), epidural progression, changes in bone density depending on the nature of metastasis and altered vascularization.

In this retrospective study, we aim to evaluate radiological changes after SBRT to osseous metastases at the last follow-up, report its oncological outcome and pain response.

## Methods

### Patient selection

**Fig. 1:**
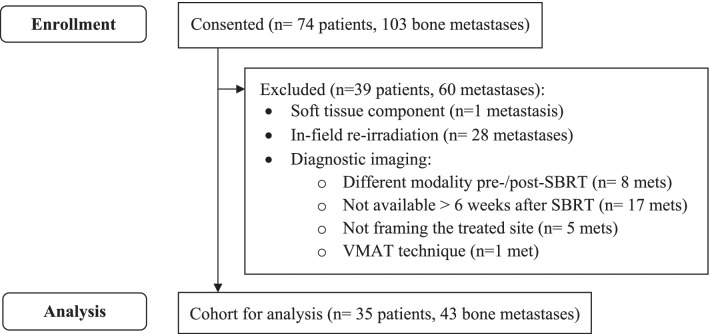
Study cohort flow diagram. Mets, bone metastases; VMAT, volumetric modulated arc therapy

After approval of the study protocol by the institutional review board and ethics committee, patient informed consent was waived. We enrolled 74 adult patients (18 years old or older) with a total of 103 spine or non-spine bone metastases, treated consecutively with SBRT between 12/2008 and 10/2018 in the radiation oncology department at Bern University Hospital, Switzerland. As shown in Fig. 1, the exclusion criteria were the following: soft tissue component (n = 1 metastasis), in-field re-irradiation (before or after SBRT, including overlapping of treatment fields) (n = 28 metastases), different diagnostic imaging modality pre-/post-SBRT (n = 8 metastases), patients with imaging less than six weeks after SBRT (n = 17 metastases), and no diagnostic images from the treated site (n = 5 metastases).

All patients had a histologic diagnosis of malignancy with either synchronous or metachronous bone metastasis. Diagnosis of bone metastasis was established by magnetic resonance imaging (MRI), computer tomography (CT), or less frequently positron emission tomography/computed tomography (PET/CT) and bone scintigraphy. Bone lesions were divided into osteolytic, osteoblastic, or mixed-form. No bone-targeted agents (i.e., denosumab, bisphosphonate, hydroxyapatite derivatives, or radioactive isotope therapy) have been administered.

### SBRT technique

Similar to what was previously described by Hwang et al. [17], the SBRT procedure consisted of 1) image acquisition, 2) patient setup and 3) SBRT planning and treatment. For planning, high-resolution thin-section MRI images were obtained (1.5 or 3 Tesla MRI). All MRI examinations included both turbo spin-echo (TSE) T1-weighted (with and without contrast enhancement) and TSE T2-weighted sequences.

For accurate and precise treatment positioning and immobilization, patients were placed in a vacuum bag (BodyFix®). The DICOM data of the MRI and CT were transferred to workstations for stereotactic planning, where the MRI was fused, at the area of interest, onto the CT images.

The target volumes for spine metastases were delineated according to international spine radiosurgery guidelines [18]. For non−spine bone metastases, a gross tumor volume/clinical target volume (GTV−CTV) margin of 3−5 mm and CTV−planning target volume of 3 mm were applied. Generally, patients with spinal metastases were treated at CyberKnife® using a spine−tracking system (SpineX®). For non−spine metastases, we have treated patients at NovalisTX using daily cone−beam CT. The median total radiation dose was 24 Gy (range 24–42 Gy) in median 3 fractions (range 2–7 fractions), prescribed to 80% isodose-line (range 70–90%).

### Follow-up imaging evaluation

As we analyze a retrospective cohort of patients, different imaging modalities have been used in order to assess the response to SBRT and evaluate the local control. Besides that, the intervals between SBRT and first follow-up visit, as well as between following visits are inhomogeneous. For example, most patients with metastatic prostate cancer were followed up using prostate-specific antigen (PSA) and the diagnostic imaging was done as the PSA raised from baseline after therapy. Despite all these inhomogeneity, we focused on MRI and CT images and observed their changes over time. This assessment was done by two independent radiologists, blind to the time of treatment. Several radiological changes were described: alterations in mineralization of sclerotic/lytic bone metastases, vertebral compression fracture for spinal metastases, pathologic fracture for non-spine metastases, morphological size progression, and signal alterations on different MRI sequences. Radiographic response assessment of metastases was classified according to University of Texas MD Anderson Cancer [19] as a complete or partial response, and stable or progressive disease, based on the last follow-up imaging.

As MRI is the most recommended imaging modality for response assessment after bone SBRT, the following changes were particularly observed on different MRI sequences: tumor volume, T2 signal intensity (SI) alterations, and contrast enhancement patterns.

Considering pre and post-treatment volumes, we categorized the tumors as decreased (group 1), unchanged (group 2), or increased (group 3). If the volumetric change was within 10% (ratio range 0.9–1.1), the lesions were regarded as unchanged in volume. In case of the absence of post-therapeutic MRI, we compared the volumes of the lesions in pre and post-therapeutic CT. T2 SI changes of the tumors were categorized into four types: (1) no changes); (2) increase in T2 signal intensity; (3) increase in T2 signal intensity intermixed with dark signal intensity, and (4) totally dark signal intensity, based on the publication from Hwang et al. [17]. Enhancement patterns were divided into two groups: no change in contrast enhancement and decrease of contrast enhancement with or without non-enhancing foci.

The SPINO consensus recommends follow-up MRI every 3–6 months after spine-SBRT; however, as mentioned before, our retrospective cohort is inhomogeneous regarding radiological follow-ups. Besides MRI, computed tomography (CT), positron emission tomography/computed tomography (PET/CT), bone scintigraphy, or single-photon emission computerized tomography (SPECT) were also performed for some patients.

### Pain response

Pain response was assessed before and at least six months after SBRT, using the numerical rating scale. We also captured the intake of painkillers and neurological deficits.

### Statistical analysis

Statistical analysis was performed with SPSS, version 22. Descriptive statistics were presented as means (M) and standard deviations (SD) for quantitative variables and frequencies (n) and percentages (%) for categorical ones.

Two-Way repeated measures ANOVA was conducted to evaluate the effect of SBRT on the quantitative parameters, along with the interaction of the effect of SBRT with the follow-up method. We also calculated the effect for the follow-up method but did not present these results due to a lack of significant values. Effect sizes were assessed with eta squared (η2), considering Cohen’s (1988) suggestion: 0.01, 0.06 e 0.14 for weak, moderate and high effect [20].

Then, we computed the difference between the two moments of assessment—i.e., the last imaging before SBRT took place and the last follow-up imaging after SBRT (ΔSBRT = (after − before SBRT)), and built linear regression models adjusting not only for the follow-up method but also for the type of lesion. This was done to reduce to number of estimated parameters and allow the computation of the effects when adjusting for these two variables. We computed unstandardized effect sizes (β), standard errors (SE) and p-values. Residual’s normality was assessed and confirmed with the Shapiro–Wilk test (p > 0.05). No residuals were found above the threshold Ri >|3|.

For assessing the categorical parameters before and after SBRT, we calculated frequencies (n), percentages (%) and Cohen’s kappa measure of agreement to assess the changes between these two moments.

Significance was considered for p < 0.05. We also considered marginally significant results for p < 0.10.

## Results

### Population

A total of 35 patients, 27 (77.1%) males and 8 (22.9%) females with 43 bone metastases were analysed in this cohort. Metastases were mainly from prostate cancer (n = 18, 51.4%), followed by breast cancer (n = 5, 14.3%). As shown in Table 1, the bony lesions are classified as spine (n = 22, 51.2%) and non-spine (n = 21, 48.8%) metastases. The spinal metastases involve mainly the lumbar spine (n = 11, 50%) and non-spinal metastases are mostly located in pelvic/hip bones (n = 15, 71.4%). The median age at the time of SBRT was 66 years old (range: 38–84).

**Table 1:** Patient, treatment and follow-up characteristics

Characteristics	Value (range)
Median follow-up, years	1.8 (< 1–8.2)
Median age at SBRT, years-old	66 (38–84)
**Dose prescription**	
Median total dose delivered, Gy	24 (24–42)
Median single dose, Gy	8 (5–12)
Median number of fractions	3 (2–7)
Median isodose prescription, %	80 (70–90)
**Imaging follow-up after SBRT**	
3 months, range	1.3–4.3
6 months, range	5.1–9.8
12 months, range	9.3–19.3

	Nr. of patients (%)
**Sex**	
Male	27 (77.1)
Female	8 (22.9)
**Histology (primary tumor)**	
Prostate	18 (51.4)
Breast	5 (14.3)
Melanoma	3 (8.6)
Non-small cell lung cancer	3 (8.6)
Other	6 (17.1)

Bone metastases location	Nr. of metastases (%)
**Non-spine**	21 (48.8)
Temporal bone	1 (4.8)
Scapula	1 (4.8)
Sternum	1 (4.8)
Rib	2 (9.5)
Pelvic/hip bones (6 ileum, 4 ischium, 4 pubis, 1 acetabulum)	15 (71.4)
Femur	1 (4.8)
**Spine**	22 (51.2)
Cervical	0 (0.0.)
Thoracic	9 (40.9)
Lumbar	11 (50.0)
Sacral	2 (9.1)

### Radiological response

Follow-up radiological assessment was performed with contrast-CT (n = 17, 39.5%), MRI (n = 26, 60.5%), and PET/CT (n = 14, 32.6%), which took place mainly three, six, and 12 months after SBRT. After a median follow-up of almost 2 years (range < 1–8.2), complete-/ and partial response, stable-/ and progressive disease occurred in 0%, 11.4%, 68.6%, and 20%, respectively. Twenty patients (57.1%) died, all from disease progression, yet 70% (n = 14) with still local stable disease after SBRT.

**Fig. 2:**
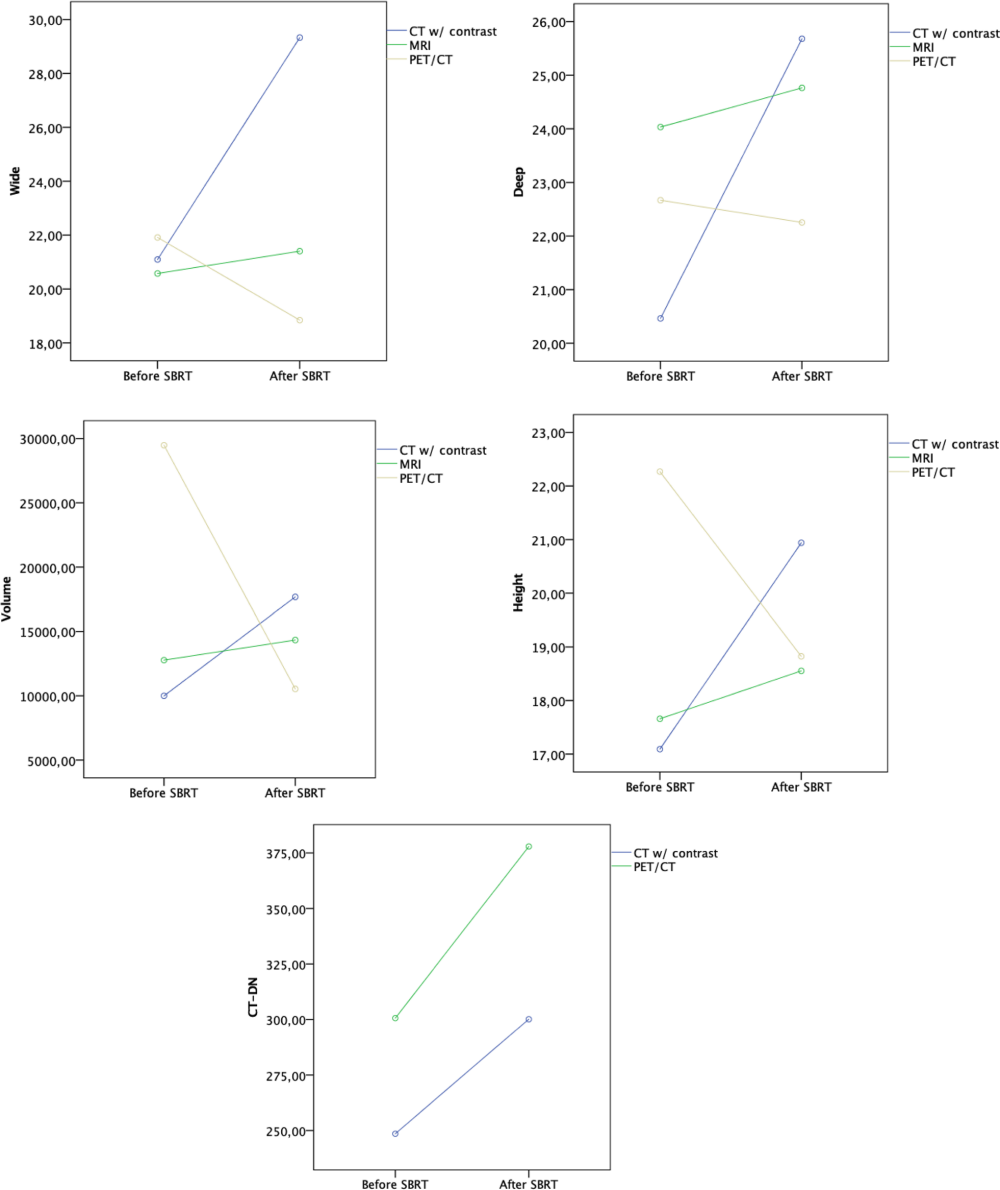
Quantitative parameters evolution and interaction with the imaging method. Abbreviations: SBRT, radiotherapy; CT w/ contrast, computed tomography contrast enhanced; MRI, magnetic resonance imaging; PET/CT, positron emission tomography/ computed tomography; CT-DN, computed tomography density native in Hounsfield units

No statistically significant difference in the radiological assessment of two independent radiologists was found. Table 2 presents results for paired comparisons regarding quantitative variables, compared by the imaging method. We found no significant differences for any of the quantitative parameters considering pre or post-SBRT. Considering interactions, we found a statistically significant difference between the width parameter and the imaging method (F = 6.13 (p = 0.004), η2 = 0.19: increased only in contrast-CT, stable in MRI, while decreased in PET/CT (Fig. 2). A marginally significant association was seen for the SBRT effect on the depth and height (respectively, F = 3.97, p = 0.052, η2 = 0.07 and F = 3.05 (p = 0.056) η2 = 0.11). There was a trend (depth: F = 3.12, p = 0.053, η2 = 0.11; height: F = 3.05 (p = 0.056) η2 = 0.11) to increase after SBRT in the contrast-CT follow-up, whereas it decreased in the PET/CT (Fig. 2). Despite the absence of significance on the volume (F = 2.23, p = 0.118), moderate effect size was found (η2 = 0.08) after SBRT, similarly with a slight increase in contrast-CT, stable results in MRI, and decrease in PET/CT. No significant or marginally significant results were found for the effect of SBRT or its interaction with the imaging method in CT density native (CT-DN). ANOVA test was not done for the CT density contrast-enhanced (CT-CE) because results were equal before and after SBRT. For the parameters T2-weighted images signal intensity (T2-SI), T2-weighted images turbo inversion recovery magnitude signal intensity (T2-TSI), T1-weighted images native signal intensity (T1-NSI), and T1-weighted images contrast-enhanced signal intensity (T1-CESI) no significant results were found for the effect of SBRT.

**Table 2:** Repeated measures ANOVA for the quantitative parameters compared by the imaging method

	CT contrast enhanced			MRI			PET/CT			Total			RM ANOVA effects	
	M	SD	n	M	SD	n	M	SD	n	M	SD	n	SBRT	Imaging
**Width**														
Pre-SBRT	21.09	11.73	15	20.58	12.74	26	21.91	14.45	14	21.91	14.45	55	F = 2.55 (p = 0.116) η2 = 0.05	F = 6.13 (p = 0.004)* η2 = 0.19
Post-SBRT	29.33	15.27	15	21.40	14.31	26	18.84	8.13	14	18.84	8.13	55		
**Depth**														
Pre-SBRT	20.46	10.26	15	24.03	13.81	26	22.67	14.65	14	22.71	13.02	55	F = 3.97 (p = 0.052)**†** η2 = 0.07	F = 3.12 (p = 0.053)**†** η2 = 0.11
Post-SBRT	25.68	10.99	15	24.76	15.72	26	22.25	11.03	14	24.37	13.30	55		
**Height**														
Pre-SBRT	17.09	6.07	15	17.66	8.28	26	22.27	17.85	14	18.68	11.07	55	F = 0.15 (p = 0.701) η2 = 0.003	F = 3.05 (p = 0.056)**†** η2 = 0.11
Post-SBRT	20.94	5.05	15	18.55	7.85	26	18.83	7.64	14	19.27	7.09	55		
**Volume**														
Pre-SBRT	9997.74	13,068.83	16	12,775.66	15,571.32	26	29,478.44	74,682.81	14	16,157.66	39,201.12	56	F = 0.42 (p = 0.502) η2 = 0.01	F = 2.23 (p = 0.118) η2 = 0.08
Post-SBRT	17,678.62	19,147.79	16	14,338.71	17,120.47	26	10,532.05	9453.02	14	14,341.30	16,164.13	56		
**CT-DN**														
Pre-SBRT	248.57	307.38	7	–	–	–	300.64	259.62	14	283.29	269.80	21	F = 2.05 (p = 0.168) η2 = 0.10	F = 0.08 (p = 0.778) η2 = 0.01
Post-SBRT	300.14	324.13	7	–	–	–	377.93	337.06	14	352.00	326.76	21		
**CT-CE**														
Pre-SBRT	263.71	323.85	7										–	
Post-SBRT	263.71	323.85	7											
**T2-SI**														
Pre-SBRT	–	–	–	224.71	175.53	19	–	–	–	–	–	–	F = 0.01 (p = 0.947) η2 = 0.00	
Post-SBRT	–	–	–	222.74	206.11	19	–	–	–	–	–	–		
**T2-TSI**														
Pre-SBRT	–	–	–	214.82	194.65	8	–	–	–	–	–	–	F = 0.06 (p = 0.816) η2 = 0.01	
Post-SBRT	–	–	–	225.63	163.17	8	–	–	–	–	–	–		
**T1-NSI**														
Pre-SBRT	–	–	–	213.76	141.40	21	–	–	–	–	–	–	F = 0.01 (p = 0.945) η2 = 0.00	
Post-SBRT	–	–	–	215.69	171.15	21	–	–	–	–	–	–		
**T1-CESI**														
Pre-SBRT				417.03	284.76	20	–	–	–	–	–	–	F = 0.01 (p = 0.945) η2 = 0.00	
Post-SBRT				357.85	267.84	20	–	–	–	–	–	–		

*p* < 0.1 is considered as significant

η2 = 0.01 weak, η2 = 0.06 moderate, η2 = 0.14 high effect, according to Cohen, J (1988). *Statistical power analysis for the behavioral sciences* (2nd ed.). Hillsdale, NJ: Erlbaum

CT, computed tomography; MRI, magnetic resonance imaging; PET/CT, positron emission tomography/ computed tomography; RM, repeated measures; M, mean; SD, standard deviation; SBRT, stereotactic body radiotherapy; CT-DN, computed tomography density native in Hounsfield units; CT-CE, computed tomography density contrast enhanced in Hounsfield units; T2-SI, T2-weighted images signal intensity; T2-TSI, T2-weighted images turbo inversion recovery magnitude signal intensity; T1-NSI, T1-weighted images native signal intensity; T1-CESI, T1-weighted images contrast enhanced signal intensity

*Statistically significant; ^†^p < 0.10

Table 3 shows the results of linear regressions for the difference before and after SBRT (ΔSBRT) adjusted for type of imaging method and type of lesion, not only showing that spinal metastases were associated with increased width (β = 4.89; p = 0.031), but also confirming that contrast-CT is associated with increased width (β = 11.82; p < 0.001), depth (β = 5.73; p = 0.017), height (β = 7.27; p = 0.012), and volume (β = 28,347.15; p = 0.026) compared with PET/CT.

**Table 3:** Linear regressions for the difference before and after SBRT adjusted for the type of imaging method and spinal lesion

	ΔSBRT = (After – Before SBRT)								
	Δ Width	Δ Depth	Δ Height	Δ Volume	Δ CT-DN	Δ T2-SI	Δ T2-TSI	Δ T1-NSI	Δ T1-CESI
**Imaging**									
CT	11.82 (3.11) p < 0.001*	5.73 (2.39) p = 0.017*	7.27 (2.90) p = 0.012*	28,347.15 (12,743.88) p = 0.026*	− 25.71 (85.02) p = 0.762	–	–	–	–
MRI	3.87 (2.76) (p = 0.162)	1.14 (2.13) p = 0.592	4.34 (2.58) p = 0.092**†**	20,438.88 (11,487.83) p = 0.075**†**	–	–	–	–	–
PET/CT	Ref	Ref	Ref	Ref	Ref	–	–	–	–
**Spine**									
Yes	4.89 (2.27) p = 0.031*	0.93 (1.74) p = 0.593	− 0.20 (2.11) p = 0.926	12,841.73 (9358.37) (p = 0.170)	− 40.56 (80.99) p = 0.617	19.16 (60.67) (p = 0.752)	− 124.38 (71.00) p = 0.080**†**	88.54 (51.23) p = 0.084**†**	112.05 (66.88) p = 0.094**†**
No	Ref	Ref	Ref	Ref	Ref	Ref	Ref	Ref	Ref

*p* < 0.1 is considered as significant

SBRT, stereotactic body radiotherapy; CT-DN, computed tomography density native in Hounsfield units; T2-SI, T2-weighted images signal intensity; T2-TSI, T2-weighted images turbo inversion recovery magnitude signal intensity; T1-NSI, T1-weighted images native signal intensity; T1-CESI, T1-weighted images contrast enhanced signal intensity; CT, computed tomography; MRI, magnetic resonance imaging; PET/CT, positron emission tomography/ computed tomography; Ref, reference

^*^Statistically significant; ^†^p < 0.10; results presented as unstandardized effect sizes β, standard errors (SE), and p-values

When we assessed the spinal metastases without adjusting for the imaging method (only data for MRI was achievable), marginally significant results were found for their association with the parameters T2-TSI (β = -124.38; p = 0.080), T1-NSI (β = 88.54; p = 0.084), and T1-CESI (β = 4.89; p = 0.031) after SBRT: lower in T2-TSI, while higher in T1-NSI and T1-CESI.

In Table 4, we present the assessment for the categorical variables’ association with pre versus post SBRT, with Cohen’s kappa measure of agreement to assess the changes between these two moments (k). CT appearance (k = 0.84), soft component (k = 0.72) and T2 turbo inversion recovery magnitude (TIRM) appearance (k = 0.67) had moderate to high agreement between the two assessments. For CT appearance, the proportion of agreement was 100% for osteolytic, 93.3% for osteoblastic and 71.4% for mixed-type lesions. For the soft tissue component, 75% agreement for presence and 94.7% for non-presence were applicable. For SI on T2-TIRM sequence, the agreement was 100% for homogenous hyper-intensity, 50% for dark spots, 50% for homogenous hypo-intensity, and 100% for the intermediary. The other parameters showed low or very low agreement, with k varying from k = 0.14 to k = 0.40.

**Table 4:** Agreement of radiological categorical variables before and after SBRT

Before SBRT	After SBRT				*k*
**CT appearance**	Osteolytic	Osteoblastic	Mixed		
Osteolytic	8 (100%)	0 (0.0%)	1 (14.3%)		0.84
Osteoblastic	0 (0.0%)	14 (93.3%)	1 (14.3%)		
Mixed	0 (0.0%)	1 (6.7%)	5 (71.4%)		
**T2 appearance**	Homogenous bright	Dark spots	Totally dark signal intensity	Intermediary	
Homogenous bright	0 (0.0%)	0 (0.0%)	1 (12.5%)	1 (25.0%)	-
Dark spots	0 (0.0%)	4 (57.1%)	0 (0.0%)	0 (0.0%)	
Totally dark signal intensity	0 (0.0%)	3 (42.9%)	7 (87.5%)	1 (25.0%)	
Intermediary	0 (0.0%)	0 (0.0%)	0 (0.0%)	2 (50.0%)	
**T2 TIRM appearance**	Homogenous bright	Dark spots	Totally dark signal intensity	Intermediary	
Homogenous bright	2 (100.0%)	1 (50.0%)	0 (0.0%)	0 (0.0%)	0.67
Dark spots	0 (0.0%)	1 (50.0%)	0 (0.0%)	0 (0.0%)	
Totally dark signal intensity	0 (0.0%)	0 (0.0%)	1 (50.0%)	0 (0.0%)	
Intermediary	0 (0.0%)	0 (0.0%)	1 (50.0%)	2 (100.0%)	
**T1 signal native appearance**	Homogenous bright	Dark spots	Totally dark signal intensity	Intermediary	
Homogenous bright	0 (0.0%)	0 (0.0%)	0 (0.0%)	0 (0.0%)	0.30
Dark spots	0 (0.0%)	1 (33.3%)	1 (7.1%)	0 (0.0%)	
Totally dark signal intensity	0 (0.0%)	2 (66.7%)	11 (78.6%)	2 (50.0%)	
Intermediary	0 (0.0%)	0 (0.0%)	2 (14.3%)	2 (50.0%)	
**T1 CE signal appearance**	No enhancement	Slight enhancement	Bright enhancement		
No enhancement	0 (0%)	0 (0%)	0 (0%)		0.14
Slight enhancement	3 (75.0%)	9 (81.8%)	3 (60.0%)		
Bright enhancement	1 (25.0%)	2 (18.2%)	2 (40.0%)		
**Contrast enhancement**	Yes	No			
Yes	17 (100.0%)	6 (66.7%)			0.40
No	0 (0.0%)	3 (33.3%)			
**Soft component**	Yes	No			
Yes	9 (75.0%)	2 (5.3%)			0.72
No	3 (25.0%)	36 (94.7%)			

SBRT, stereotactic body radiotherapy; CT, computed tomography; T2 TIRM, T2-weighted

Regarding toxicity, we identified one (4.6%) VCF in spine metastasis and another new/progressive fracture in one (4.8%) non-spine metastasis.

### Pain response

We observed that 22 (62.9%) patients remained asymptomatic on the treated metastases. Four (11.4%) patients reported decreased pain (complete and partial); three (8.6%) had stable pain (two without regular analgesia and one stable after SBRT but requiring analgesia after surgery); two (5.7%) complained of increased pain, even though one of them without requiring analgesia. Therefore, from patients who were symptomatic and available for follow-up, almost half (44.4%) reported pain reduction after SBRT. Four patients (11.4%) had no available pain response follow-up, whereas one of them deceased before it was captured. None presented neurological deficits before or after SBRT.

## Discussion

Results of the current retrospective study present SBRT as an effective treatment for bone metastases. SBRT has been increasingly accepted as a valuable option for selected patients with metastatic disease. Using the appropriate imaging modality for treatment planning, SBRT offers excellent local control with an acceptable toxicity profile [21]. However, response assessment after SBRT is a challenging topic, which is not only limited to the bone, as we confront difficulties to interpret the changes in imaging modalities after stereotactic radiotherapy in other organs, like the brain, liver and lung [22–24]. Appropriate evaluation of diagnostic images is a critical point in the process of the disease and can prevent the risks of unnecessary interventions. Besides that, pain response should be considered after radiotherapy for bone metastases, as pain relief is the most important goal in such patients. In this retrospective study, we report our institutional results regarding imaging-based local control and pain response after SBRT for bone metastases, independently of the systemic therapy used. Despite not being captured in this analysis, as the main histologies were prostate and breast cancer, hormonal therapy, and chemotherapy must have been often applied. Other limitations are the small sample size of 35 patients and the varied radiation dose regimen applied.

The SPINO group published a report in 2015, focusing on response assessment after SBRT for spinal metastases [13]. The consensus is based on an international survey and not yet evaluated in clinical trials. We considered the recommendations from SPINO group for image-based tumor and pain response, although we analyzed both spine and non-spine bone metastases in our study. The MRI is the preferred modality for response assessment after SBRT; however, we should be aware of some unique aspects such as pseudo-progression and VCF when interpreting the post-SBRT images.

**Fig. 3:**
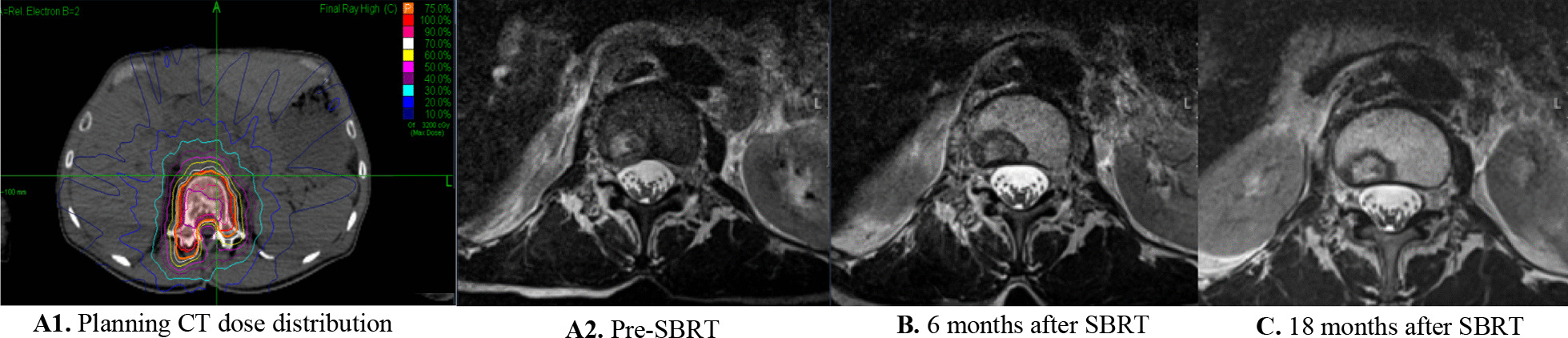
Example of radiological changes of a spine metastasis (**A2**) treated with SBRT (stable disease (**C**), yet initially classified as “pseudoprogression” (**B**)), and associated SBRT-plan (**A1**)

Pseudo-progression is a well-known phenomenon after SBRT in different organs. It was first reported for spine metastasis in a case report from 2015 where the authors described it as a subacute, post-radiotherapy reaction that mimics progressive disease (PD) with increased contrast enhancement and ultimate stabilization and regression [25]. Time is an important factor evaluating post-SBRT radiological changes, as pseudo-progression presents a few weeks up to 6 months after radiation, in contrast to radio-necrosis which is a late effect and can occur even years after therapy [25]. Amini et al. did an analysis of osseous pseudo-progression in the vertebral body following SBRT in patients from two prospective phases I/II clinical trials [26]. They defined the osseous pseudo-progression as “transient growth in signal abnormality centered at the lesion with a sustained decline on follow-up MRI that was not attributable to chemotherapy”. They reported the rates of pseudo-progression and PD of 14% and 24%, respectively. Furthermore, there was a significant association between single fraction SBRT and the development of pseudo-progression [26]. The so far published randomized trials comparing SBRT versus conventional radiotherapy have not reported the rates of pseudo-progression [12, 27, 28]. The SPINO group defined any new or progressive tumor within the epidural space as local progression [13], but we have recently published a case report showing clear epidural involvement on radiological images after spine SBRT, yet histologically without tumor cells in the epidural space [29]. Therefore, it is critical to distinguish between pseudo-progression, PD and radio-necrosis to avoid false patient management. In our study, we observed pseudo-progression as a common finding after SBRT (Fig. 3); however, not all patients had MRI shortly after the therapy and therefore it was not possible to report the exact rate of pseudo-progression.

VCF is a well-known and most common complication after spine SBRT. The rate of VCF after single fraction SBRT with 18–24 Gy was reported around 39%, and lytic lesions and location below T10 confer a high risk of fracture [30]. The median time to fracture was 25 months and VCF was seen earlier in patients with lytic lesions compared to sclerotic lesions [30]. Sahgal et al. reported 14% of new or progressing VCF after spine SBRT, using different fractionation and considering SINS-score to determine its predictive value [31]. They defined the high dose per fraction, lytic lesion and baseline fracture as significant predictors of VCF [31]. A review from 2017 reported a crude VCF rate of 13.9% [32]. Jawad et al. demonstrated low rates of VCF for 5.7% in their multi-institutional study, using 1–5 fractions for spine SBRT [33]. We report here the rates of new/progressive fractures for spine (i.e., VCF) and non-spine metastases as 4.6% and 4.8%, respectively. As half of our cohort had metastatic prostate cancer, one reason for our low rates of fractures could be the sclerotic nature of the metastases. Another reason might be related to our moderate SBRT schema with a median total dose of 24 Gy in three fractions.

As mentioned above, MRI is the most recommended imaging modality for radiological assessment of bone metastasis after SBRT. Hwang et al. reported the MRI changes after SBRT for osteoblastic spinal lesions, as these metastases usually show no obvious radiological volumetric alterations [17]. They classified signal intensity (SI) alterations on T2-MRI sequences as follows: (1) no changes in SI; (2) increased SI; (3) increased SI intermixed with dark SI; (4) changed to complete dark SI. Most of our patients had prostate cancer as primary diagnosis; therefore, we assessed the T2 weighted MRI sequences for radiological response evaluation after SBRT for both spine and non-spine bone metastases as described above. According to recommendations from the SPINO group, the routine use of contrast-enhanced T1-MRI sequences to visualize spinal metastases is controversial as both normal bone marrow and tumor are enhanced [13]. The interpretation becomes even more difficult after SBRT and therefore we considered the T1-MRI with gadolinium only for delineating the epidural and para-spinal tumor components. Although the patient population was heterogeneous in our cohort, SBRT achieved 80% of LC at almost 2 years. More than 40% of our patients survived and among the population who died in follow-up time, 40% had still SD at the irradiated sites. These results are in line with data from other studies, showing an excellent rate of LC after SBRT for osseous metastases [21].

Considering pain response, the randomized phase 2 trial from Germany reported significantly improved pain values in SBRT group 6 months after the therapy in patients with spinal metastases [12]. However, as they chose the single fraction SBRT with 24 Gy, the rates of new pathological fracture were high in that study, with 8.7% and 27.8% at 3 and 6 months respectively [12]. Another randomized phase 2 trial from the Netherlands compared SBRT versus conventional radiotherapy for bone metastases using different fractionations [28]. SBRT group did not show significant pain improvement, but because of selective dropout, this trial was underpowered to detect the difference in pain response [28]. The NRG Oncology/RTOG 0631 trial initial results were presented at ASTRO annual meeting in 2019 [34]. Randomizing patients with spinal metastases into SBRT and conventional radiotherapy groups, this study showed negative results for the SBRT arm, as pain control was similar at three months between the two groups. Finally, the Canadian randomized phase 3 trial compared spine SBRT with 24 Gy in two daily fractions with conventional RT at a dose of 20 Gy in five fractions [27]. The SBRT was superior to conventional radiotherapy and improved the complete pain response at three months. Interestingly, the incidence of VCF was equal between the two groups, showing the safety of the SBRT regimen [27]. In our retrospective study, the majority of patients (62.9%, n = 22) had no pain prior to SBRT and the indication was mostly local ablation in oligo-metastatic/progressive disease. This group of patients remained asymptomatic after SBRT. In the symptomatic group (25.7%, n = 9), only two patients experienced pain exacerbation following SBRT with only one of them requiring analgesic medication.

The next step should be to conduct a prospective study, with larger sample size and a fixed-dose protocol, comparing different imaging modalities with response assessment, especially for solid tumors with specific tumor biomarkers, such as functional MRI or prostate-specific membrane antigen PET/CT (for prostate cancer).

## Conclusion

Spine and non-spine metastases SBRT can achieve high rates of tumor and pain control. Local control was analyzed in different imaging modalities. Further comparative studies are warranted in a prospective, standardized manner.

## Data Availability

The datasets used and/or analyzed during the current study are available from the corresponding author on reasonable request.

## Abbreviations

SBRT: Stereotactic body radiation therapy
SD: Stable disease
PD: Progressive disease
LC: Local control
OS: Overall survival
VCF: Vertebral compression fracture
MRI: Magnetic resonance imaging
CT: Computed tomography
PET: Positron emission tomography
TSE: Turbo spin echo
PSA: Prostate specific antigen
SI: Signal intensity
NCI: Native signal intensity
CESI: Contrast-enhanced signal intensity
TSI: Turbo inversion recovery magnitude signal intensity
SPECT: Single photon emission computer tomography
SE: Standard error
DN: Density native
CE: Contrast enhanced
TIRM: Turbo inversion recovery magnitude
